# Effects of timing on intracoronary autologous bone marrow-derived cell transplantation in acute myocardial infarction: a meta-analysis of randomized controlled trials

**DOI:** 10.1186/s13287-017-0680-5

**Published:** 2017-10-16

**Authors:** Jia-ying Xu, Dai Liu, Yang Zhong, Rong-chong Huang

**Affiliations:** 1 Department of Cardiology, The First Affiliated Hospital of Dalian Medical University, 222 Zhongshan Road, Dalian, 116011 People’s Republic of China; 2 Present address: Department of Cardiology, The Fifth People’s Hospital of Dalian City, Dalian, People’s Republic of China

**Keywords:** Acute myocardial infarction (AMI), Cell therapy, Cells, Bone marrow-derived cells (BMCs), Meta-analysis

## Abstract

**Background:**

Several cell-based therapies for adjunctive treatment of acute myocardial infarction have been investigated in multiple clinical trials, but the timing of transplantation remains controversial. We conducted a meta-analysis of randomized controlled trials to investigate the effects of timing on bone marrow-derived cell (BMC) therapy in acute myocardial infarction (AMI).

**Methods:**

A systematic literature search of PubMed, MEDLINE, and Cochrane Evidence-Based Medicine databases from January 2000 to June 2017 was performed on randomized controlled trials with at least a 3-month follow-up for patients with AMI undergoing emergency percutaneous coronary intervention (PCI) and receiving intracoronary BMC transfer thereafter. The defined end points were left ventricular (LV) ejection fraction, LV end-diastolic and end-systolic index. The data were analyzed to evaluate the effects of timing on BMC therapy.

**Results:**

Thirty-four RCTs comprising a total of 2,307 patients were included; the results show that, compared to the control group, AMI patients who received BMC transplantation showed significantly improved cardiac function. BMC transplantation 3–7 days after PCI (+3.32%; 95% CI, 1.91 to 4.74; *P* < 0.00001) resulted in a significant increase of left ventricular ejection fraction (LVEF). As for the inhibitory effect on ventricular remodeling, BMC transplantation 3–7 days after PCI reduced LV end-diastolic indexes (–4.48; 95% CI, −7.98 to –0.98; *P* = 0.01) and LV end-systolic indexes (–6.73; 95% CI, –11.27 to –2.19; *P* = 0.004). However, in the groups who received BMC transplantation either within 24 hours or later than 7 days there was no significant effect on treatment outcome. In subgroup analysis, the group with LVEF ≤ 50% underwent a significant decrease in LV end-diastolic index after BMC transplantation (WMD = –3.29, 95% CI, –4.49 to –2.09; *P* < 0.00001); the decrease was even more remarkable in the LV end-systolic index after BMC transplantation in the group with LVEF ≤ 50% (WMD = –5.25, 95% CI, –9.30 to –1.20; *P* = 0.01), as well as in patients who received a dose of 10^7–10^8 cells (WMD = –12.99, 95% CI, –19.07 to –6.91; *P* < 0.0001). In the group with a follow-up of more than 12 months, this beneficial effect was significant and increased to a more pronounced effect of +3.58% (95% CI, 1.55 to 5.61; *P* = 0.0006) when compared with control.

**Conclusions:**

In this meta-analysis, BMC transfer at 3 to 7 days post-AMI was superior to transfer within 24 hours or more than 7 days after AMI in improving LVEF and decreasing LV end-systolic dimensions or LV end-diastolic dimensions. It is more effective in patients with lower baseline LVEF (≤50%) and the effect can last more than 12 months.

**Electronic supplementary material:**

The online version of this article (doi:10.1186/s13287-017-0680-5) contains supplementary material, which is available to authorized users.

## Background

With progress in intervention techniques and drug treatment, the mortality of acute myocardial infarction (AMI) has been significantly reduced. However, the incidence of heart failure after myocardial infarction remains high [1]. Therefore, ways to restore heart function after myocardial infarction and to increase the long-term survival rate is the major research concern. Strauer et al*.* [2] performed autologous bone marrow-derived cell (BMC) transplantation on AMI patients for the first time, confirming the safety and effectiveness of stem cell transplantation. A quantity of basic and clinical studies on transplantation of BMCs in AMI treatment have produced mixed results as the subjects, the approach, the timing and the type and dose of transplanted stem cells varied. Existing meta-analyses of BMC transplantation in AMI focuses on the safety and effectiveness of BMC transplantation in AMI patients [3–5] and there have been few studies concerned with the specific approach used for BMC transplantation and evaluation.

Basic studies show that after AMI, increases in the expression of inflammatory chemokines and other cytokines might enhance the homing and differentiation of BMCs. In contrast, a strong inflammatory reaction often accompanies the release of reactive oxides and other cytokines at the site of infarction after AMI, which is detrimental to the survival and differentiation of autologous myocardial cells and transplanted BMCs [6, 7]. In addition, BMCs transplanted at different stages are affected by the microenvironment of myocardium. This may influence the survival and differentiation of transplanted BMCs, and may even cause their apoptosis [8, 9]. Therefore, the timing of BMC transplantation is considered to be one of the crucial factors affecting the survival of transplanted BMCs and hence the effectiveness of treatment.

A meta-analysis of different timings of intracoronary transplantation of BMCs in AMI patients was performed to study the effect of timing of stem cell transplantation on AMI treatment. Our research will provide a clue for the choice of optimal timing of BMC transplantation in AMI treatment.

## Methods

### Study source and selection

Literature on randomized controlled trials (RCTs) of autologous BMC treatment of AMI from January 2000 to June 2017 was retrieved from PubMed, MEDLINE and Cochrane databases. The subject terms or keywords used to retrieve the literature were “cell therapy, cell transplantation, stem cell therapy, bone marrow-derived cell, acute myocardial infarction”. Study selection was performed by two different researchers.

Studies were included that met the following criteria: (1) type of research: RCTs; follow-up duration: at least 3 months; (2) object of research: clinically diagnosed as AMI. The cases in the experimental group received percutaneous coronary intervention (PCI) and BMC transplantation. The cases in the control group did not receive bone marrow-derived stem cells (BMSCs) (e.g. control media or plasma); (3) intervention therapy: the cases in the experimental group received intracoronary transplantation of autologous BMCs, regardless of the type and dose of stem cells administered; (4) outcome indicators: primary indicators included left ventricular ejection fraction (LVEF), left ventricular end-diastolic volume (LVEDV) (or left ventricular end-diastolic volume index, LVEDVI) and left ventricular end-systolic volume (or left ventricular end-systolic volume index, LVESVI); secondary indicator was a cardiovascular adverse event; (5) publication in English or Chinese. The exclusion criteria were as follows: (1) intravenous injection or intramyocardial injection; (2) cytokine intervention or stem cell mobilization using cytokines; (3) lack of control group. Differences in researchers’ assessments of articles were resolved by a discussion with a third researcher.

### Data extraction

Data extraction and analysis was performed by three independent researchers. For each study, we documented the articles and they were categorized into trial characteristics and functional outcome. Different assessments from data extraction were resolved by a discussion with a third researcher. Study information was recorded as follows: study design, baseline characteristics of included study, risk of bias of each study.

### Quality assessment

The quality of RCTs was assessed by The Cochrane Collaboration’s tool for assessing risk of bias (See Additional file 1: Figure S1).

### Definition

A variety of stem cells included in this studies, such as bone marrow-derived cells, bone marrow stem cells, bone marrow mononuclear cells and bone marrow progenitor cell. BMCs were the main focus of this study because the majority of studies to date assessed this specific cell type. The outcome of LV function (e.g. LVEF), left ventricular end-diastolic index (e.g. LVEDV, LVEDVI), as well as left ventricular end-systolic index (e.g. LVESV and LVESVI) were the primary end points of our analysis, and mainly measured by cardiac magnetic resonance imaging (MRI), echocardiography, positron emission tomography-computed tomography (PET-CT) and single-photon emission computed tomography (SPECT).

### Statistical analysis

The variations of left ventricular functional indexes in experimental and control groups compared with baselines were analyzed using Revman 5.3 software (The Cochrane Collaboration, Copenhagen, Denmark). We studied the difference in mean change (from baseline to follow-up) between patients who received stem cells and those who received control treatment. Most outcomes were reported as mean ± SD at baseline and follow-up. The mean change was then determined as follow-up to baseline, whereas SD change was determined as SEM (sample size). However, weighted mean difference (WMD) and standardized mean difference (SMD) were both expressed as a 95% confidence interval (CI).

The χ^2^ test was implemented to test the heterogeneity among the included trials: we considered that at *P* values ≤ 0.10, the trials could be considered heterogeneous. Therefore, trials that might give rise to heterogeneity were excluded and the reasons for such heterogeneity were identified. A funnel plot was drawn to evaluate possible bias. Subgroup analysis was performed to calculate summary statistics. If heterogeneity still existed after such treatment, the random effect model was employed to calculate summary statistics by the D-L method (DerSimonian and Laird method) [10]. I^2^ was used to quantify the heterogeneity. If I^2^ > 50%, the heterogeneity was considered significant.

Subgroup analysis investigated the effect on summary statistics brought about by the timing of stem cell transplantation (within 24 hours or between 3 and 7 days or over 7 days), baseline LVEF (≤50% or > 50%), follow-up time(<6 months,< 12 months or ≥ 12 months) and dose of BMCs administered.

## Results

### Search results

**Fig. 1:**
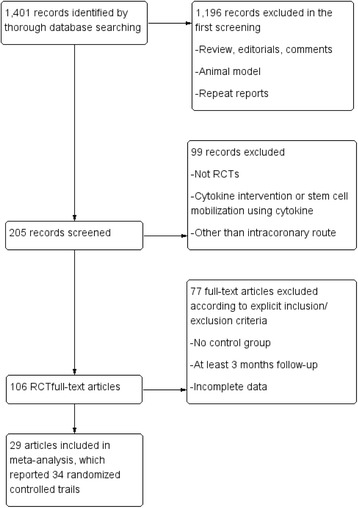
Flow diagram process of data collection and screening

Preliminary retrieval obtained 1,401 papers, of which 1,196 were excluded by reading the title and abstract. The excluded papers were concerned with animal trials, comments, reviews and repeatedly reported trials. The remaining 205 papers were read thoroughly and 99 summaries or papers concerned with nonrandomized controlled trials were excluded. Another 77 papers were also excluded due to incomplete data, no setting up of a control group or use of a BMC injection method other than intracoronary injection. Finally, 29 articles were included in the meta-analysis, which reported 34 RCTs, comprising a total of 2,307 patients, 1,112 of whom were treated with an injection of cells (Fig. 1).

### Study characteristics

General information of included studies: after screening, 29 articles [11–39] were included in the meta-analysis, reporting data from a total of 34 RCTs comprising a total of 2,307 patients, 1.112 of whom were treated with cells. All of the included trials contained a control group and a BMC transplantation group. The average age of the patients was 55.7 ± 2.8 years, and male patients accounted for 84.6 ± 8.3%. Baseline LVEF was (44.7 ± 7.3%); intracoronary injection of autologous BMCs was performed using a dose of (7.17 ± 16.4) × 10^8 cells. Other information included the follow-up duration and the timing of BMC injection (Table 1).

**Table 1:** Characteristics of studies included in the meta-analysis

Source	Sample size	Male (%)	Age (year)	Primary intervention and route of delivery	Cell type	Cell dose	Time of cell therapy	Follow-up (months)	Measure tools	Baseline LVEF (%)
Janssens 2006 [11]	67	82	56.9	PCI IC	BMSC	304 × 10^6	Within 1 day after PCI	4	MRI	48.1
Yousef 2009 [12]	62	89.6	51.1	PCI IC	BMC	6.1 (3.9) × 10^7	Mean 7 days after PCI	60	Echo	51.2
Wang 2014 [13]	58	60.6	57	PCI IC	BMSC	1 × 10^8	Mean 15 days after PCI	6	Echo	29
Piepoli 2010 [14]	38	68.4	65	PCI IC	BMC	418 × 10^6	3 to 5 days after PCI	12	Echo	37.1
Panovsky (HD) 2008 [15]	47	89.3	54	PCI IC	BMC	1 × 10^8	Mean 7 days after PCI	3	SPECT	35
Panovsky (LD) 2008 [15]	47	89.3	54	PCI IC	BMC	1 × 10^7	Mean 7 days after PCI	3	SPECT	35.5
Gao 2013 [16]	54	93.2	56.8	PCI IC	BMSC	3.08 (0.52) × 10^6	Mean 17 days after PCI	12	SPECT,Echo	51.1
Sürder (day 6) 2016 [17]	150	84.1	57.7	PCI IC	BMSC	1.40 × 10^8	Mean 6 days after PCI	12	MRI	37.1
Sürder (day 24) 2016 [17]	150	84.1	57.7	PCI IC	BMSC	1.60 × 10^8	Mean 24 days after PCI	12	MRI	37.6
Traverse 2011 [18]	87	82.8	56.1	PCI IC	BMC	150 × 10^6	2 to 3 weeks after PCI	6	MRI	47
Plewka 2009 [19]	60	73	56	PCI IC	BMSC	1.44 (0.49) × 10^8	Mean 7 days after PCI	6	Echo	39.5
Traverse (day 3) 2012 [20]	67	88	56.3	PCI IC	BMC	150 × 10^6	3 days after PCI	6	MRI	43.9
Traverse (day 7) 2012 [20]	53	87.2	57.6	PCI IC	BMC	150 × 10^6	7 days after PCI	6	MRI	46.4
Chen 2004 [21]	69	95.5	57.5	PCI IC	BMSC	8 × 10^9	Nearly 18 days after PCI	6	SPECT	48.5
Ge 2006 [22]	20	90	58.5	PCI IC	BM-MNC	4 × 10^7	Within 1 day after PCI	6	SPECT,Echo	56
Wöhrle 2013 [23]	42	-	-	PCI IC	BMC	324 × 10^6	Mean 7 days after PCI	36	CMR	54.6
Herbots 2009 [24]	67	82.1	56.5	PCI IC	BMPC	304 (128) × 10^6	Within 1 day after PCI	4	MRI	54.4
Grajek 2010 [25]	45	86.7	50.4	PCI IC	BMSC	2.34 (1.2) × 10^9	4 to 6 days after PCI	12	Echo	50.6
Dill 2009 [26]	54	90.7	56.3	PCI IC	BMC	236 (174) × 10^6	3 to 6 days after PCI	12	CMR	47.8
Wollert 2004 [27]	60	70	56.3	PCI IC	BMC	24.6 × 10^8	4.8 days after PCI	6	MRI	50.7
Roncalli 2011 [28]	101	85.3	55.5	PCI IC	BMC	98.3 (8.7) × 10^6	7 to 10 days after PCI	3	RNA	36.3
Meyer 2009 [29]	60	70	56.3	PCI IC	BMC	24.6 (9.4) × 10^8	Mean 4.8 days after PCI	61	MRI	50.7
Schächinger 2006 [30]	204	82	56	PCI IC	BMC	2.36 (1.74) × 10^8	Within7days after PCI	4	LVangiography	47.6
Bartunek 2005 [31]	35	91	54	PCI IC	BMPC	12.6 (2.2) × 10^6	Mean 11.6 days after PCI	4	SPECT	44.7
Lunde 2006 [32]	100	84	57.4	PCI IC	BMC	68 × 10^6	Mean 6 days after PCI	6	SPECT	42
Beitnes 2011 [33]	100	84	57.4	PCI IC	BM-MNC	68 × 10^6	Mean 6 days after PCI	36	Echo	46.3
Huang 2006 [34]	40	67.5	57	PCI IC	BM-MNC	1.8 (4.2) × 10^8	Within 1 day after PCI	6	CMR	44
Cao 2009 [35]	86	93.2	56.8	PCI IC	BM-MNC	5 (1.2) × 10^7	7 days after PCI	48	Echo	38.8
Hopp 2011 [36]	28	78.5	58.6	PCI IC	BMC	8.71 × 10^7	Mean 6 days after PCI	6	CMR	51.7
Yao 2009 [37]	39	84.6	52	PCI IC	BMC	1.9 (1.2) × 10^8	3 to 7 days after PCI	12	MRI	32.4
Meluzín (HD) 2008 [38]	60	91.7	54.3	PCI IC	BMC	10^8	Mean 7 days after PCI	12	SPECT	40
Meluzín (LD) 2008 [38]	60	91.7	54.3	PCI IC	BMC	10^7	Mean 7 days after PCI	12	SPECT	40.5
Wollert (LD) 2017 [39]	64	87.2	53.8	PCI IC	BMSC	7.00 × 10^8	Mean 8.1 days after PCI	6	MRI	45.7
Wollert (HD) 2017 [39]	59	88.1	56.1	PCI IC	BMSC	20.60 × 10^8	Mean 8.1 days after PCI	6	MRI	45.8

*BMC* bone marrow-derived cells, *BMSCs* bone marrow stem cells, *BM*-*MNC* bone marrow mononuclear cells, *BMPC* bone marrow progenitor cell, *PCI* percutaneous coronary intervention, *IC* intracoronary, *Echo* echocardiography, *MRI* magnetic resonance imaging, *SPECT* single-photon emission computed tomography, *LVEF* left ventricular ejection fraction

In this analysis, 2,307 patients had a complete set of baseline and follow-up LVEF measurements (34 RCTs), 1,622 patients had complete left ventricular end-diastolic indexes measurements (26 RCTs) and 1,447 patients had complete left ventricular end-systolic indexes measurements (23 RCTs).

### Effect of time window of BMC transplantation on left ventricular function

**Fig. 2:**
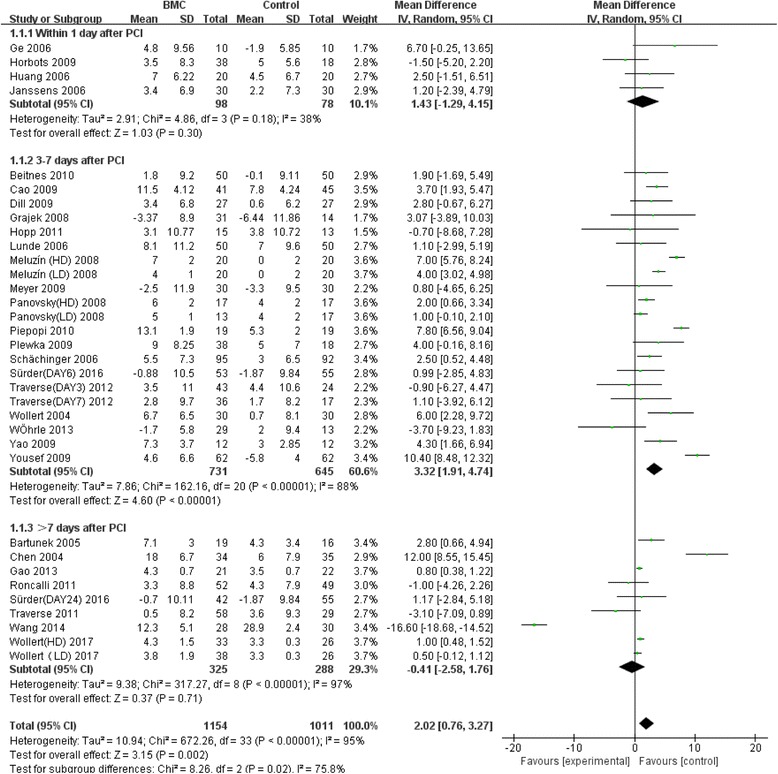
Effect of timing of BMC transplantation on left ventricular ejection fraction

**Fig. 3:**
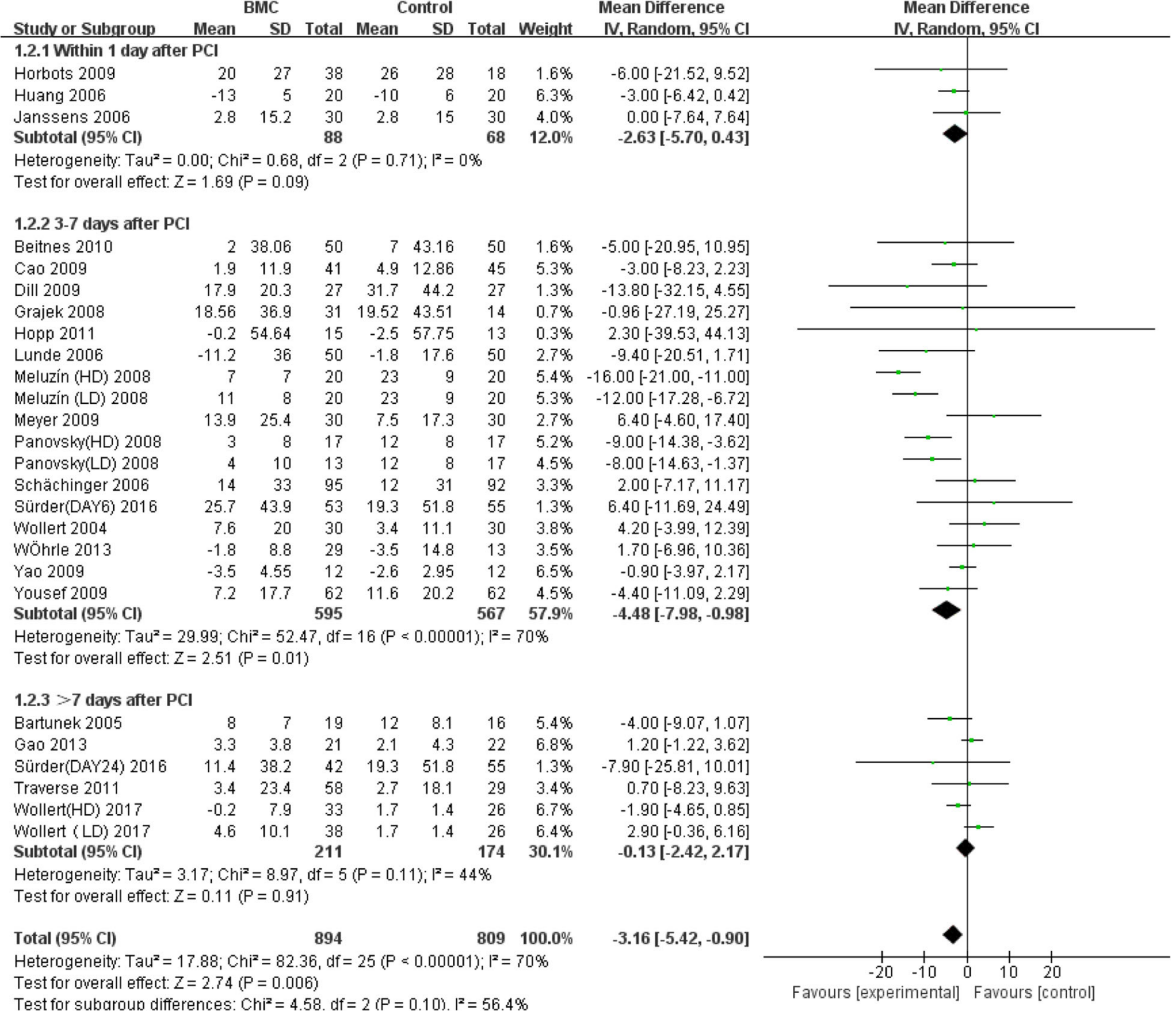
Effect of BMC transplantation timing on left ventricular end-diastolic indexes

**Fig. 4:**
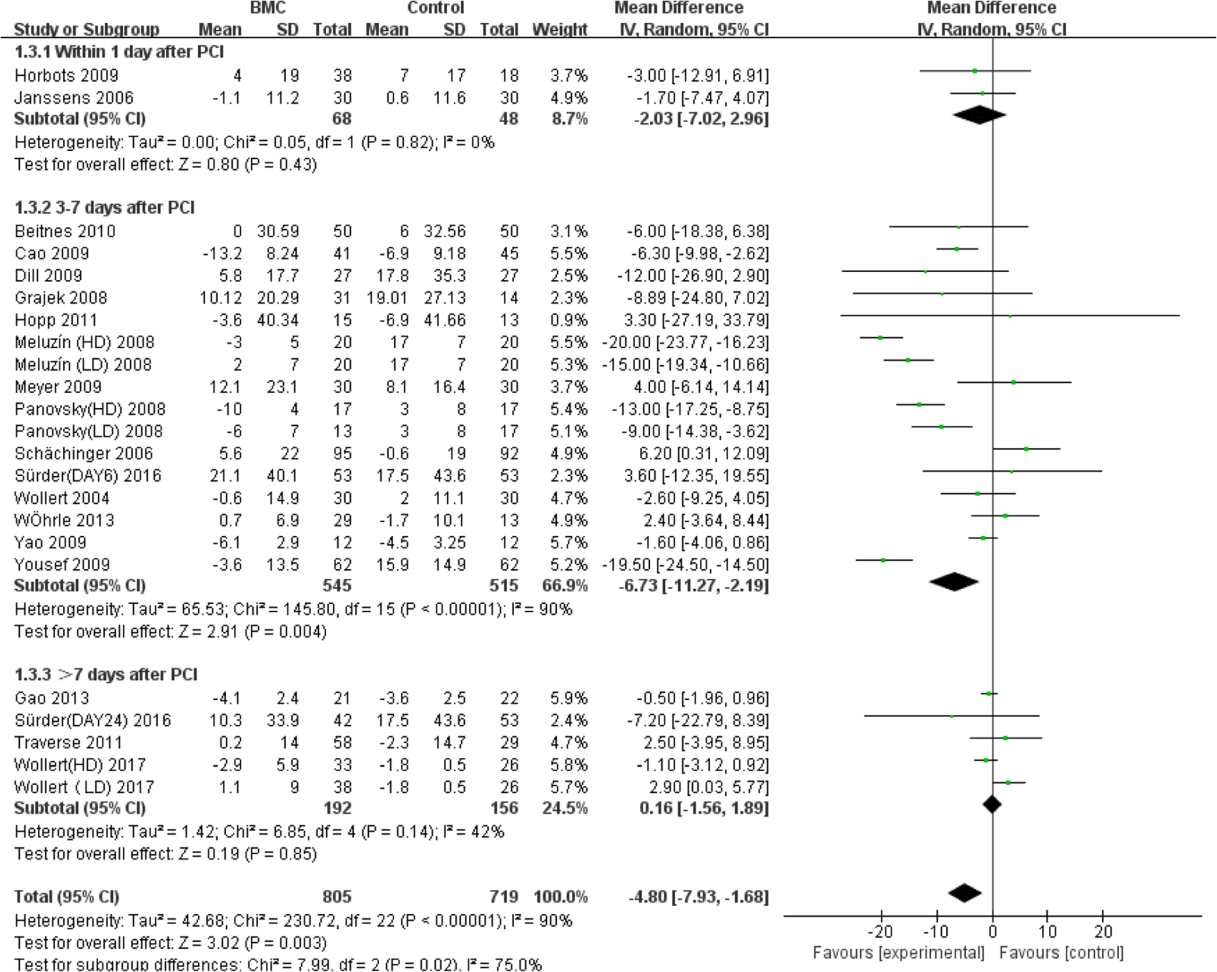
Effect of timing of BMC transplantation on left ventricular end-systolic indexes

Meta-analysis of the included studies was conducted and summary statistics were calculated. The experimental groups and control groups which received stem cell transplantation within 1 day, 3–7 days and over 7 days after percutaneous coronary intervention were compared. Indicators were the variations of LVEF and left ventricular end-diastolic and end-systolic indexes compared with baselines, in the evaluation of the effect of stem cell transplantation on cardiac functions and on inhibition of left ventricular remodeling. The results of our analysis indicated that overall LVEF is increased by 2.02% (95% CI, 0.76 to 3.27; *P* = 0.02). The LVEF of the BMC transplantation group with a time window of 3–7 days was significantly increased by 3.32% (95% CI, 1.91 to 4.74; *P* < 0.00001) (Fig. 2). The forest plot shows that the unadjusted difference in the mean change in LVEDI with a time window of 3–7 days significantly decreased by 4.48 mL (95% CI, −7.98 to –0.98; *P* = 0.01) in the BMC group (Fig. 3). Further, LVESI with a time window of 3–7 days was significantly decreased by 6.73 mL (95% CI, −11.27 to −2.19; *P* = 0.002) in the treatment group (Fig. 4). These results showed much improved left ventricular functions in the BMC transplantation group with a time window of 3–7 days compared with the groups with time windows of up to 24 hours or over 7 days.

### Subgroup analysis

Left ventricular function was significantly improved in the BMC transplantation group with a time window of 3–7 days. However, significant heterogeneity (I^2^ > 50%) still existed among the studies. Therefore, subgroup analysis was conducted of the baseline LVEF values and the follow-up period used, which were possible sources of heterogeneity. The trials were divided into groups as follows: baseline LVEF values > 50% and ≤ 50%; duration of follow-up < 6 months, < 12 months and ≥ 12 months; and cell doses of ≤ 10^7, ≤ 10^8, ≤ 10^9 or ≤ 10^10 BMCs.

### Effect of baseline LVEF before BMC transplantation on left ventricular function

The group with LVEF ≤ 50% (18 RCTs) underwent a significant decrease in LV end-diastolic index after BMC transplantation (WMD = –3.29, 95% CI, –4.49 to –2.09, *P* < 0.00001); the decrease in LVESI after BMC transplantation was even more remarkable in this group (15 RCTs) (WMD = –5.25, 95% CI, –9.30 to –1.20, *P* = 0.01, Table 2). However in both groups, baseline LVEF did not have a significant effect on treatment outcome in terms of LV ejection fraction. According to our data, patients with a higher LVEF (>50%) at baseline did not benefit more from cell therapy compared with patients with lower LVEF (≤50%).

**Table 2:** Effect of baseline LVEF before BMC transplantation on left ventricular function

	No. of RCTs	Difference in mean (95% CI)	*P* value
LV ejection fraction			
Baseline LVEF > 50%	9	2.60 [–1.06 to 6.26]	0.16
Baseline LVEF ≤ 50%	25	1.79 [0.22 to 3.37]	0.03
LV end-diastolic index			
Baseline LVEF > 50%	8	0.93 [–1.13 to 2.99]	0.38
Baseline LVEF ≤ 50%	18	–3.29 [–4.49 to –2.09]	< 0.00001
LV end-systolic index			
Baseline LVEF > 50%	8	–3.72 [–10.19 to 2.75]	0.26
Baseline LVEF ≤ 50%	15	–5.25 [–9.30 to –1.20]	0.003

*RCTs* randomized controlled trials, *95% CI* 95% confidence interval

### Effect of follow-up period after BMC transplantation on left ventricular function

Subgroup analysis revealed that in patients with follow up periods of < 6 months (7 RCTs), LVEF increased by 1.45% (95% CI, 0.54 to 2.36; *P* = 0.002, Table 3). In the group with a follow-up period of ≥ 12 month (14 RCTs), this beneficial effect was significant and increased to a more pronounced effect of 3.58% (95% CI, 1.55 to 5.61; *P* = 0.0006, Table 3) when compared with controls. Both the group with a follow-up duration of < 6 months and that with follow-up of ≥ 12 months underwent a significant decrease in LVEDI after BMC transplantation (WMD = –4.76, 95% CI, –8.19 to –1.33; *P* = 0.006; WMD = –4.00, 95% CI, –8.13 to 0.13; *P* = 0.06, Table 3); the decrease in LVESI was more sustained in the group with a follow-up duration of ≥12 months (WMD = –7.07, 95% CI, –11.99 to–2.14; *P* = 0.005, Table 3). These data demonstrate that cell transplantation in patients with AMI can result in an improvement which is not only short-term, and that this effect can last more than 12 months.

**Table 3:** Effects of BMC transplantation over time

	No. of RCTs	Difference in mean (95% CI)	*P* value
LV ejection fraction			
Follow up < 6 months	7	1.45 [0.54 to 2.36]	0.002
Follow up > 6, < 12 months	13	0.83 [–1.85 to 3.51]	0.54
Follow up ≥ 12 months	14	3.58 [1.55 to5.61]	0.0006
LV end-diastolic indexes			
Follow up < 6 months	6	–4.76 [–8.19 to –1.33]	0.006
Follow up > 6, < 12 months	7	–0.60 [–3.38 to 2.18]	0.67
Follow up ≥ 12 months	13	–4.00 [–8.13 to 0.13]	0.06
LV end-systolic indexes			
Follow up < 6 months	5	–4.29 [–11.51 to 2.92]	0.24
Follow up > 6, < 12 months	5	0.51 [–1.92 to 2.95]	0.68
Follow up ≥ 12 months	13	–7.07 [–11.99 to –2.14]	0.005

*RCTs* randomized controlled trials, *95% CI* 95% confidence interval

### Effect of cell dose used for BMC transplantation on left ventricular function

Intracoronary infusion of BMCs (cell dose of ≤ 10^8) resulted in a significant decrease in LVEDI by 7.36 mL (95% CI, –11.45 to–3.27; *P* = 0.0004, Table 4), whereas BMC transplantation resulted in a decrease in LVESI by 12.99 mL (95% CI, –19.07 to –6.91; *P* < 0.0001, Table 4). LVEF increased by 1.56% after transplantation of BMCs (95% CI, –2.95 to 6.06; *P* = NS; Table 4). Subgroup analysis revealed the tendency that the effect of BMC transplantation maybe better in patients who received a dose of 10^7–10^8 bone marrow cells.

**Table 4:** Effect of cell dose used for BMC transplantation left ventricular function

	No. of RCTs	Difference in mean (95% CI)	*P* value
LV ejection fraction			
Cell dose ≤ 10^7	3	1.91 [–0.05 to 3.87]	0.06
Cell dose > 10^7, ≤ 10^8	11	1.56 [–2.95 to 6.06]	0.50
Cell dose > 10^8, ≤ 10^9	16	1.65 [0.22 to 3.09]	0.02
Cell dose > 10^9, ≤ 10^10	4	5.87 [0.79 to 10.95]	0.02
LV end-diastolic indexes			
Cell dose ≤ 10^7	3	–6.00 [–15.20 to 3.20]	0.20
Cell dose > 10^7, ≤ 10^8	8	–7.36 [–11.45 to –3.27]	0.0004
Cell dose > 10^8, ≤ 10^9	12	–0.69 [–2.22 to 0.83]	0.37
Cell dose > 10^9, ≤ 10^10	3	4.63 [–1.74 to 11.00]	0.15
LV end-systolic indexes			
Cell dose ≤ 10^7	3	–8.01 [–17.88 to 1.87]	0.11
Cell dose > 10^7, ≤ 10^8	6	–12.99 [–19.07 to –6.91]	< 0.0001
Cell dose > 10^8, ≤ 10^9	11	0.32 [–1.62 to 2.26]	0.75
Cell dose > 10^9, ≤ 10^10	3	–1.50 [–6.90 to 3.89]	0.59

*RCTs* randomized controlled trials, *95% CI* 95% confidence interval

### Adverse events

**Fig. 5:**
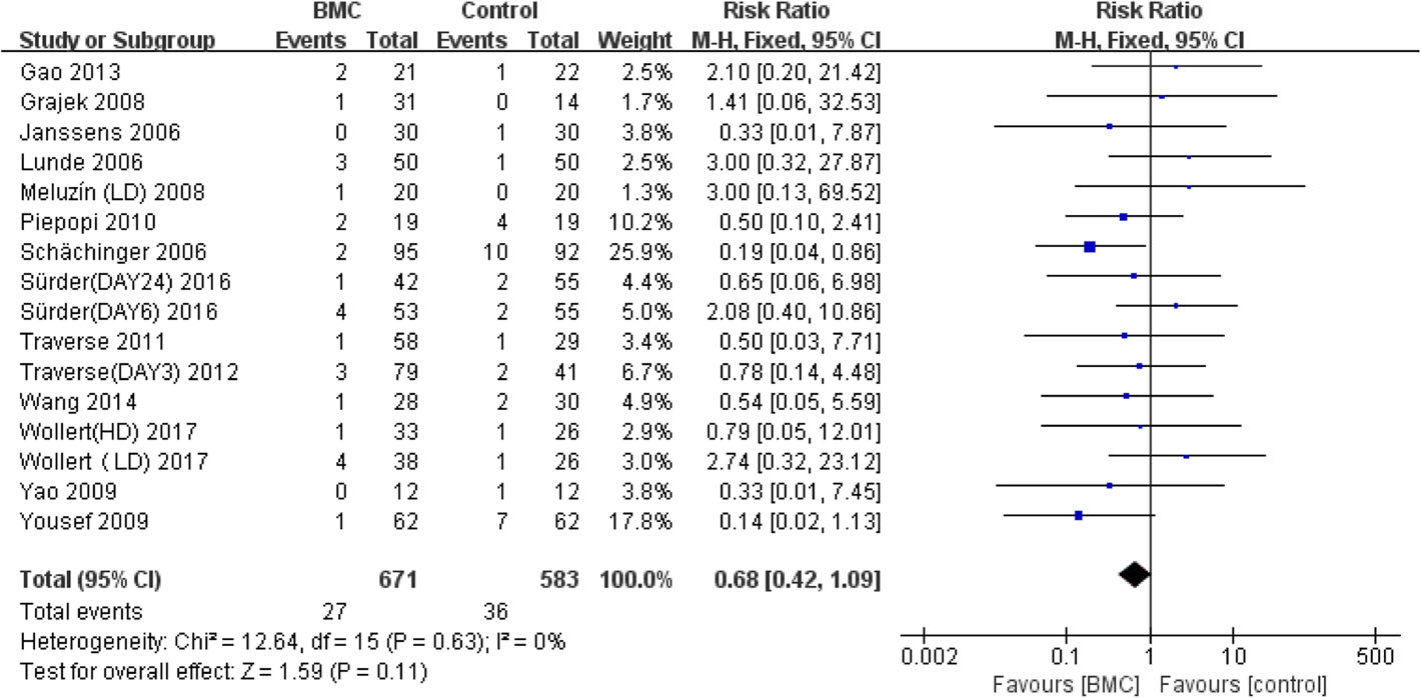
Mortality and reinfarction of stem cell transplantation

The safety of BMC transplantation is also important at the moment. We chose death and reinfarction of the patients. Our study found that the stem cells transplantation had not too much influence on mortality and reinfarction (Fig. 5).

### Sensitivity analysis and publication bias

**Fig. 6:**
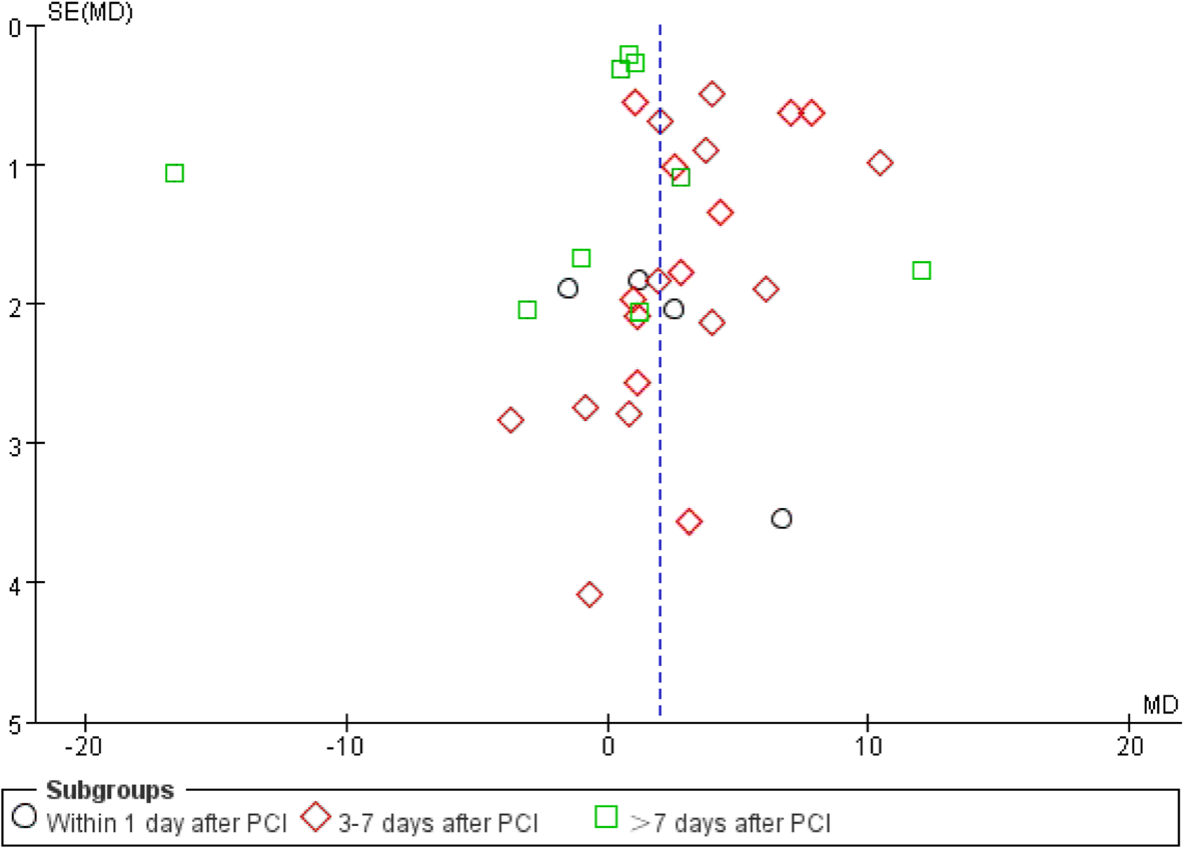
Funnel plot of timing of BMC transplantation on left ventricular ejection fraction

Sensitivity analysis is a method of evaluating the stability and reliability of meta-analyses. By getting rid of each trial one by one, the sensitivity analysis evaluates the stability of the WMD of LVEF and is used to observe whether summary statistics change. A funnel plot of LVEF values showed that studies were equally distributed around the overall estimate, suggesting that there was no evidence of publication bias (Fig. 6).

## Discussion

In this meta-analysis, which comprises a total of 2,307 patients with AMI, cell therapy proved to be safe. Previous meta-analyses on autologous BMC transplantation in AMI patients were mostly concerned with the security and effectiveness of such a technique. Therefore, the present research placed a greater emphasis on the effect of timing of stem cell transplantation and the choice of optimal time window.

Our study showed that compared to the control group, AMI patients demonstrated significantly improved cardiac function after receiving BMC transplantation. BMC transplantation 3–7 days after AMI resulted in a significant increase of LVEF. As for the inhibitory effect against ventricular remodeling, BMC transplantation 3–7 days after PCI reduced LVEDI and LVESI. However, in both the group who underwent transplantation within 24 hours and those who underwent transplantation at over 7 days, no significant effect was observed on treatment outcome. The statistics showed much improved left ventricular function in the BMC transplantation group with a time window of 3–7 days compared to those with a time window of < 24 hours or over 7 days. This result showed that inflammation may influence the bone marrow-derived cells to settle and differentiate in the early phase (within 24 hours) of acute myocardial infarction; when BMC transplantation occurs over 7 days after PCI, the fibroblasts present and the mechanical traction exerted by scar tissue during this period also have an impact on the ability of BMCs to differentiate. Consequently, choosing the right time to perform the transplantation not only directly relates to the survival of the stem cells, but also relates to the biological effects of the stem cells, and thus to the improvement in cardiac function.

Subgroup analysis found that the effect of BMC transplantation LVEF has no difference in the 10^7-10^8 bone marrow cells, but LVEDVI and LVESVI show a clearly improve in the 10^7-10^8 range. Some studies found that 10^8 is the lowest cell number to achieve favorable effects on LV function [40–42]. However, Wang et al. showed that the injection of no more than 10^7 MSCs for AMI after percutaneous coronary intervention might improve left ventricular systolic function [43]. So we need more research to clarify which dosage is better.

At the same time, our study demonstrates that cell transplantation in patients with AMI can result in a significant elevation of LVEF after a follow-up duration of both < 6 month and > 12 month, but not at < 12 month. This may associate with the incidence of adverse events, the inflammatory response, or time of stem cell differentiation and so on. So further study is needed to clarify this.

According to our data, baseline LVEF, dose of BMCs used and duration of follow-up might all influence the effectiveness of BMC treatment. Experiments by Janssens et al*.* [11] and Tendera et al*.* [44] found that the effect of BMC transplantation was more apparent in patients with low LVEF. However, the recent HBEB [45] and BONAMI studies [28] with a large sample size found that cardiac function of patients after a larger area of AMI or lower baseline LVEF (≤45%) were not significantly improved after BMC transplantation compared to the control group (38.6% ± 24.7% vs. 42.4% ± 18.7%; *P* = 0.33). Our research showed that the improvement was more significant in patients whose baseline LVEF values were ≤ 50%.

Regarding the dose of BMCs administered, it is believed that the dose correlates positively with the improvement in cardiac function, i.e., the greater the number of bone marrow-derived cells reaching the ischemic and infarct zone, the greater the effectiveness will be. However, due to differences in the animal models used, type of transplanted cells, and cell isolation and purification techniques between different studies, the dose of cells administered varied greatly from one study to another. A large number of studies were devoted to dose dependency. Iwasaki et al. [46] found that the function of myocardium and angiogenesis in mice was dose-dependent when CD34+ cells were injected to infarcted myocardium. These findings indicate that transplantation of a higher dose of cells is more effective than low doses in the treatment of damaged myocardium. Our study also showed that the effectiveness of BMC transplantation was better in patients who received a dose of 10^7–10^8 bone marrow cells.

The first clinical long-term studies showed a gradually increasing functional benefit of cell transplantation within the first post-transplant year [47]. In contrast, Meluzín et al*.* [38] found that autologous mononuclear bone marrow cell transplantation could significantly improve left ventricular systolic function after AMI, but benefit of cell transplantation was partially lost during the 12-month follow-up. In this meta-analysis we found that cell transplantation in patients with AMI can not only provide short-term improvement, but that this effect can last more than 12 months.

### Limitations

Although the results proved the effectiveness of BMC transplantation in treatment of AMI by meta-analysis of randomized controlled trials involving a large number of subjects, and no publication bias was found, the present study also had its limitations. Currently, the individual patient data (IPD) meta-analysis is the gold standard for meta-analyses; it can assess the impact of a treatment on clinical outcomes, especially in the case of small and medium-sized clinical studies. The IPD database is kept simple; therefore, a meta-analysis cannot evaluate some surrogate parameters if data are not gathered or factors are not available, such as different quality of life assessment scores.

Heterogeneity was quite apparent among included trials, and might arise from the non-homogeneity in the baseline indicators of subjects, dose of cells used for stem cell transplantation, timing of stem cell transplantation, the choice of placebo for the control groups, and measurement and outcome indicators. In addition, the duration of the follow-up period differed between different trials; some trials involved a medium-term or long-term follow-up. This might result in uncertainty of the long-term effectiveness of BMC transplantation. Therefore, more basic and clinical studies are required to examine the possible mechanisms of BMC transplantation in treating AMI and to standardize the approach to BMC transplantation.

The difficulties of the standard meta-analysis approaches have been reviewed here. Each has a place in the analysis of data when pivotal clinical trials are not available and each sheds light on the magnitude of the treatment effect in a complex health-care field.

## Conclusions

In conclusion, meta-analysis of 29 papers reporting the results of 33 randomized controlled trials confirmed that BMC transplantation could significantly improve cardiac function after AMI, and that it could also increase LVEF and prevent ventricular remodeling. BMC transplantation 3–7 days after PCI was more effective than that within 24 hours or at over 7 days. Subgroup analysis indicated that BMC transplantation was more effective in patients with lower baseline LVEF values when cells were administered at a dose of 10^7–10^8 BMCs, and that this effect could last more than 12 months.

## Additional file

Additional file 1: Figure S1. Risk of bias summary: each risk of bias item for each included study (DOCX 180 kb)

## Abbreviations

AMI: Acute myocardial infarction
BMC: Bone marrow-derived cell
CI: Confidence interval
IPD: individual patient data
LVEDV: Left ventricular end-diastolic volume
LVEDVI: Left ventricular end-diastolic volume index
LVEF: Left ventricular ejection fraction
LVESV: Left ventricular end- systolic volume
LVESVI: Left ventricular end- systolic volume index
MRI: Magnetic resonance imaging
PCI: Percutaneous coronary intervention
PET-CT: Positron emission tomography-computed tomography
RCT: Randomized controlled trials
SEM: Sample size
SMD: Standardized mean difference
SPECT: Single-photon emission computed tomography
WMD: Weighted mean difference
