# Supplementary figures and images for: Effects of timing on intracoronary autologous bone marrow-derived cell transplantation in acute myocardial infarction: a meta-analysis of randomized controlled trials
Source: Stem Cell Res Ther. 2017 Oct 16;8:231. doi: 10.1186/s13287-017-0680-5 (PMC5644258; doi:10.1186/s13287-017-0680-5)

**Figure S1.** Risk of bias summary: each risk of bias item for each included study


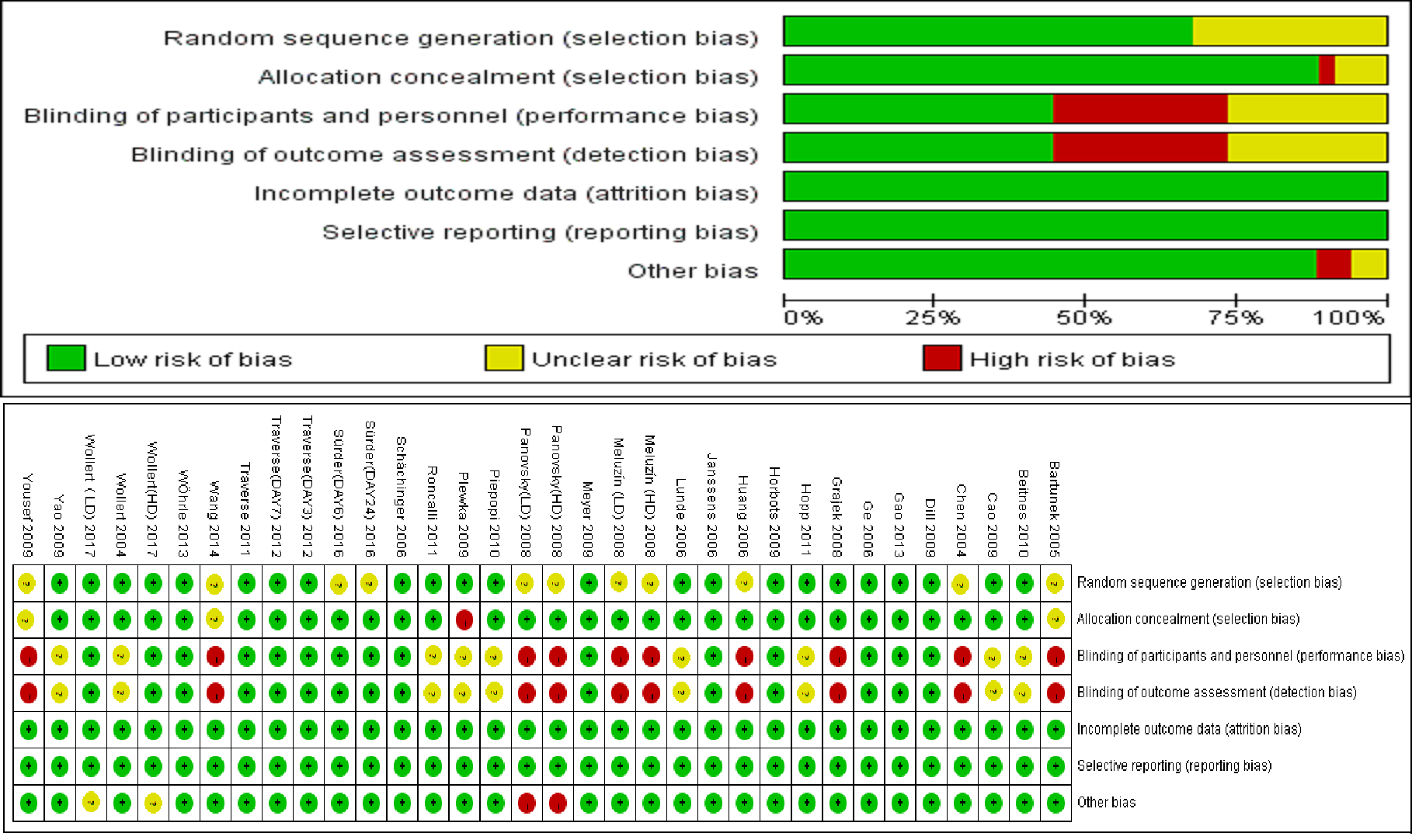

Supplement: Additional file 1: Figure S1. — Risk of bias summary: each risk of bias item for each included study (DOCX 180 kb) [file 13287_2017_680_MOESM1_ESM.docx]
